# Cytotoxicity and Radiosensitizing Potentials of Pilosulin-3, a Recombinant Ant Venom, in Breast Cancer Cells

**DOI:** 10.3390/toxins15120701

**Published:** 2023-12-15

**Authors:** Reema M. Alzeer, Khaled S. Al-Hadyan, Najla M. Al-Harbi, Sara S. Bin Judia, Rafa S. Almeer, Ghazi A. Alsbeih

**Affiliations:** 1 Department of Zoology, College of Science, King Saud University, Riyadh 11541, Saudi Arabia; 2 Biomedical Physics Department, Radiation Biology Section, King Faisal Specialist Hospital & Research Centre, Riyadh 11211, Saudi Arabia

**Keywords:** Pilosulin-3, ant venom, radiosensitization, cytotoxicity, breast cancer

## Abstract

Venom peptides are promising agents in the development of unconventional anticancer therapeutic agents. This study explored the potential of Pilosulin-3, a recombinant peptide from the venom of the Australian jack jumper ant “*Myrmecia pilosula*”, as a cytotoxic and radiosensitizing agent in MCF-7 and MDA-MB-231 breast cancer (BC) cell lines. Pilosulin-3’s cytotoxicity was evaluated across a wide range of concentrations using a proliferation assay. Cell cycle progression and apoptosis were examined at the inhibitory concentration 25% (IC25) and IC50 of Pilosulin-3, both with and without a 4Gy X-ray irradiation dose. Radiosensitivity was assessed at IC25 using the clonogenic survival assay. The study revealed that Pilosulin-3 exerted a concentration-dependent cytotoxic effect, with IC25 and IC50 values of 0.01 and 0.5 µM, respectively. In silico screening indicated high selectivity of Pilosulin-3 peptide, which was predicted to be the most likely anticancer agent (PROB = 0.997) with low hemolytic activity (PROP = 0.176). Although Pilosulin-3 exhibited a significant (*p* < 0.05) G2/M cell cycle arrest in combination with radiation, there was no discernible effect on apoptosis induction or cell survival following irradiation. In conclusion, Pilosulin-3 proved to be cytotoxic to BC cells and induced a cytostatic effect (G2/M arrest) when combined with radiation. However, it did not enhance the efficacy of cell killing by irradiation. While it holds potential as a cytotoxic agent in breast cancer treatment, its application as a radiosensitizer does not find support in these results.

## 1. Introduction

Cancer is a life-threatening disease exhibiting complex hallmarks of malignant transformation that culminate in sustaining uncontrollable cell growth, escaping the immune surveillance system and displaying altered responses to stimuli and treatment [1]. The leading malignancies affecting both men and women, constituting over half of all cases, include lung, breast, colorectal, prostate, stomach, and liver cancers [2]. Based on the World Health Organization’s (WHO) 2020 statistics, collated by the International Agency for Research on Cancer (IARC), female breast cancer became the most diagnosed cancer globally, with 2.3 million cases detected in 2020, surpassing, for the first time, the number of new cases of lung cancer [3]. Breast cancer accounts for 11.7% of all new cancer cases [3].

Scientific research has made significant progress in understanding breast cancer, resulting in a significant reduction in mortality [4]. Conventional chemoradiation cancer treatments can have significant side effects, prompting scientists to search for more natural agents that can cure cancer without harming normal cells in the body [5,6]. Typically, most drug-discovery efforts in natural products have focused on plants; however, insects, which are widely diverse throughout the world, are also important sources of active molecules with potential untraditional anticancer potential [7]. Active compounds have been isolated from ants, wasps, bees, cockroaches, flies, and other insects [8,9]. These natural insect products have shown various biological activities, including antimicrobial, anticancer, antioxidant, antiviral, anti-inflammatory, and immunomodulatory effects [7]. Insects’ venoms are capable of activating mammalian sensory neurons to produce pain and can also incapacitate arthropods, indicating dual utility in both defense and predation [10]. As a result, they offer a wide repertoire of active ingredients for medicinal use [11].

The Australian jack jumper ant (*Myrmecia pilosula*) is one of the frequently studied ant species, particularly with regard to its venom composition and bioactivity [12]. The venom is composed of various enzymes and four families of highly basic, low-molecular-weight peptides known as Pilosulins [13]. These peptides are unique and share little structural homology with other venom peptides from *Hymenoptera* [14]. They are relatively simple, with predominant peptides making up about 90% of the venom [13]. Pilosulins have been shown to exhibit antimicrobial activities, cytotoxicity, histamine-releasing properties, and hypotensive effects [13]. The four main peptides, namely, Myr p 1 (pilosulin-1), Myr p 2 (which includes Pilosulin-3 and delta-Myrtoxin-Mp1a), Myr p-3 (Pilosulin-4), and Pilosulin-5 (a histamine-releasing peptide), have been recognized as allergens by the International Union of Immunology Societies [15]. All four peptides are highly basic; however, within *M. pilosula* venom, Pilosulin-3 has been identified as a major allergen [15,16]. Inagaki et al. [17] have characterized Pilosulin-3 as a 74-amino-acid peptide that has two cysteine residues and a glycine at the C-terminal, rich in hydrophobic and basic amino acids. They found that it caused a significant and dose-dependent histamine release from rat peritoneal mast cells at concentrations as low as 5 µM, and that it displayed antimicrobial activity against some bacteria species, as well as low hemolytic activities.

It is well-known that ant stings can cause inflammation, erythema, pruritus, vesicles, and sterile pustules, which can lead to allergic reactions and even anaphylactic shock [18,19]; nevertheless, there is a cultural belief that ant venoms could help with inflammatory disorders such as arthritis, psoriasis, and others [20]. In fact, most published studies have focused on the allergenic properties of ant venoms [21]. In contrast with other insect species, there is limited information available about the characteristics of ant venom proteins as anticancer agents [22,23]. Paradoxically, despite the known allergic responses, a traditional belief persists that venom might possess potential anticancer properties. This belief fuels a growing interest in exploring these venoms for their possible therapeutic benefits in cancer treatment. Enhancing the efficacy of current treatments and finding novel approaches are of great importance in cancer therapy. A few reviews were published that gathered the available information about the use of venoms as an anticancer agent [22,23,24]. Venom peptides can arguably induce cell death in cancer cells and can substantially enhance the efficacy of chemotherapy and radiotherapy [24]. However, the venom-origin of the anticancer drug has yet to be achieved because we still lack of clear understanding of the anticancer action [23]. This is despite the identification of candidate mechanisms of venom-based peptides such as inducing apoptotic cell-killing, halting cancer cell proliferation, invasion, angiogenesis, and metastasis, which are among the characteristic hallmarks of cancer that can be used in therapy [22].

Significant progress has been made in the development and treatment of breast cancer, with multi-modal care options including surgery, radiation therapy, chemotherapy, immunotherapy, and hormonal therapy [25]. Given that the traditional use of venoms often involves cytolytic activity, it is tempting to speculate that ant venom could be a promising area of research for the design of novel anticancer drugs [26]. Most studies on Pilosulin-3 and related ant venoms have dealt with its allergic, hemolytic, and antimicrobial activities [13]. However, its effects on cancer cells remain unexplored. This study examines the cytotoxic and radiosensitizing potential of Pilosulin-3 in breast cancer cell lines. The study explores the potential effects of Pilosulin-3 on cell cycle progression, apoptosis induction, and cell survival in combination with X-ray radiation.

## 2. Results

### 2.1. Specificity of Pilosulin-3 Peptide

The ENNAACT online program was used to screen for specificity and selectivity of Pilosulin-3 toward normal and cancer cells. The full length of the mature (residues 51–73) peptide sequence (IIGLVSKGTCVLVKTVCKKVLKQ) was utilized for prediction purposes. The normalized sigmoid scores (PROP) yielded a value of 0.176 for hemolytic activity and 0.997 for anticancer activity.

### 2.2. Cell Proliferation Using the Real-Time Cell Analyzer (RTCA)

**Figure 1:**
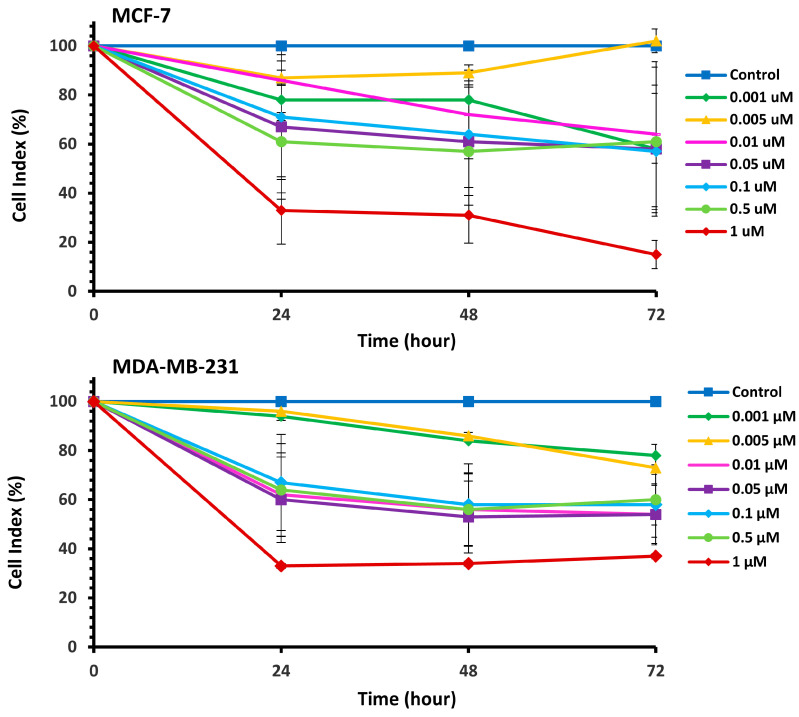
The normalized cell index (CI %) effect of Pilosulin-3 on BC cell lines. Data points represent the average CI ± SD.

The cytotoxic effects of Pilosulin-3 on breast cell lines were assessed using the RTCA cell proliferation assay (Supplementary Figure S1). The assay depicts the evolution of cell index (CI) values over time in response to incubation with varying concentrations of Pilosulin-3 in MCF-7 and MDA-MB-231 cell lines. Pilosulin-3 was added to the cells at 24 h (−/+ 1 h) to allow for cell attachment to the surface of the RTCA plate. The CI of MCF-7 (doubling time = 27 h) increased steadily until about 72 h and then reached a plateau. In contrast, the CI of MDA-MB-231 (doubling time = 18 h) increased until about 36 h and then gradually decreased up to 96 h. To determine the effect of Pilosulin-3 on cell proliferation, the RTCA data, following the addition of Pilosulin-3, were normalized by dividing the CI of treated wells by that of the untreated control wells (0 µM). The results are shown in Figure 1, where increasing concentrations of Pilosulin-3 led to a proportional decrease in CI in both cell lines. The inhibitory effect of Pilosulin-3 appeared to peak at 24 h and continued up to 72 h. At the highest concentration (1 µM), the CI (%) decreased to 15% (standard error (SE) = 5.73) and 37% (SE = 2.41) at 72 h in MCF-7 and MDA-MB-231, respectively.

**Figure 2:**
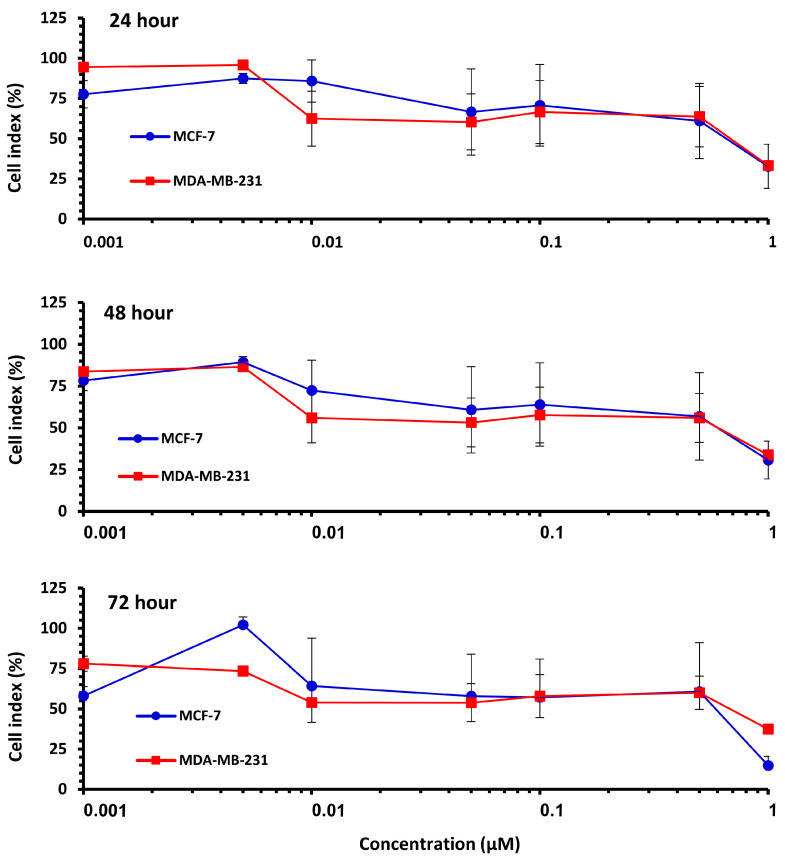
The effect of Pilosulin-3 on BC cell lines for each time point. Colored curve represents MCF-7 and MDA-MB-231. Data points represent the average CI ± SD.

The effect of Pilosulin-3 concentration on CI at 24 h, 48 h, and 72 h is illustrated in Figure 2. Both cell lines showed similar patterns of effect, with a decrease in CI corresponding to increasing Pilosulin-3 concentration. To determine the inhibitory concentrations (IC), the data points were pooled and fitted to a linear regression. The concentrations of Pilosulin-3 required to reduce CI by 50% and 25% (IC50 and IC25) were calculated to be 0.5 µM and 0.01 µM, respectively. These IC50 and IC25 values were used in subsequent experiments.

### 2.3. Pilosulin-3 Effect on Cell Cycle Progression

**Figure 3:**
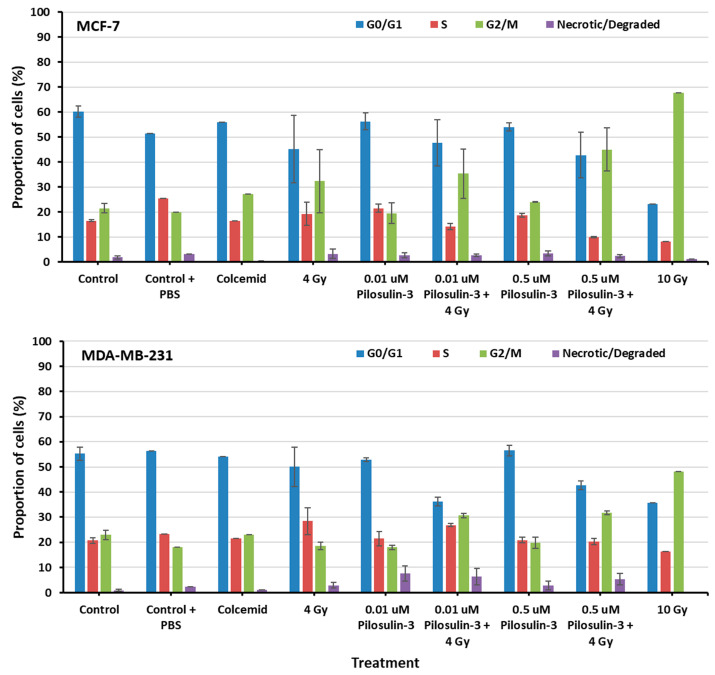
The flow cytometry analysis of cell cycle progression of MCF-7 and MDA-MB-231 cells. The percentages of cells in each phase of the cell cycle were plotted as indicated. Data represent the mean ± SE of three independent experiments. Statistical analysis showed a significant overall difference in G2/M among the various treatments (*p* = 0.02 and 0.001 for MCF-7 and MDA-MB-231, respectively). Pair-wise comparison highlighted significant differences between the control and the combined treatments (*p* < 0.05).

The effect of Pilosulin-3 on cell cycle progression was assessed using flow cytometry (Supplementary Figure S2). As shown in Figure 3, cells under control conditions displayed a typical distribution of cell cycle phases in both breast cancer cell lines (average of 58% in G0/G1, 19% in S, 22% in G2/M, and 1% as necrotic/degraded cells). The addition of colcemid relatively increased the proportion of cells in G2/M in MCF-7 (27.11%) but not in MDA-MB-231 (23.13%). Irradiation caused a dose-dependent increase in G2/M, especially in MCF-7 compared to MDA-MB-231 (32.32% and 18.62% at 4 Gy, 67.68% and 48.16% at 10 Gy, respectively). Pilosulin-3 had no obvious effect on cell cycle progression as a single agent. However, the combination of drug (0.01 and 0.5 µM) and irradiation (4 Gy) showed a trend toward an increase in the percentages of cells in G2/M phase beyond that observed with irradiation alone, especially at the highest concentration (45.01% and 31.74% in MCF-7 and MDA-MB-231, respectively). Statistical analysis using one-way repeated-measures analysis of variance showed that there is an overall significant difference (*p* = 0.02 and *p* = 0.001 for MCF-7 and MDA-MB-231, respectively) in the percentage of cells in G2/M between the different treatment groups. In particular, the pair-wise comparison indicates a statistically significant difference between the control and the combined treatment (*p* < 0.05).

### 2.4. Pilosulin-3 Effect on Apoptosis

**Figure 4:**
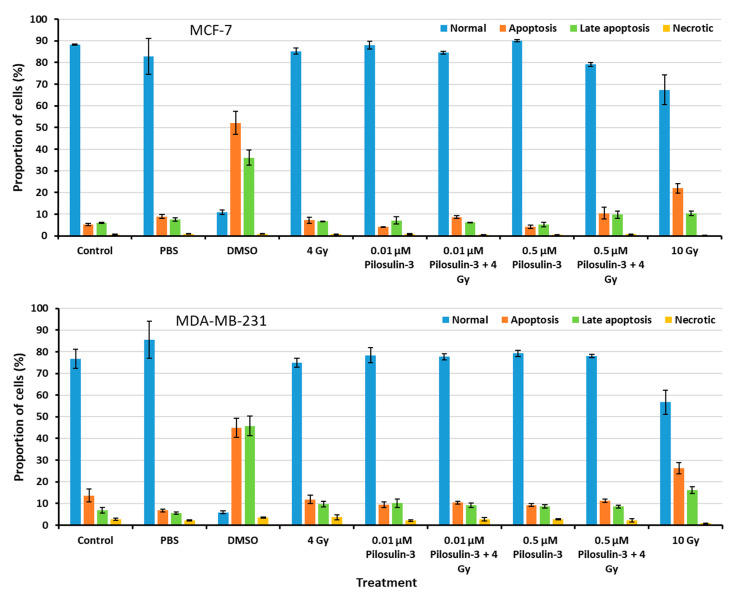
Flow cytometric analysis of apoptosis in MCF-7 and MDA-MB-231 cells. The percentages of cells in each phase of the cell cycle were plotted. Data represent the mean ± SE of three independent experiments.

The effect of Pilosulin-3 on apoptosis induction in breast cancer cells was assessed using flow cytometry (Supplementary Figure S3). As shown in Figure 4, the proportion of cells in the normal, apoptosis, late apoptosis, and necrosis phases were similar for all treated cells compared to the negative control in both cell lines. These results indicate that Pilosulin-3 has no effect on cell apoptosis as a single agent or in combination with drug-radiation (4 Gy) at the concentrations used in the current study. DMSO, which was used as a positive control, induced apoptosis in MCF-7 and MDA-MB-231 cells at rates of 52.14% (SE = 5.21) and 44.88% (SE = 4.49), respectively. Likewise, the highest radiation dose, 10 Gy, induced apoptosis in MCF-7 and MDA-MB-231 cells at rates of 21.95% (SE = 2.19) and 26.35% (SE = 2.63), respectively.

### 2.5. Clonogenic Cell Survival

**Figure 5:**
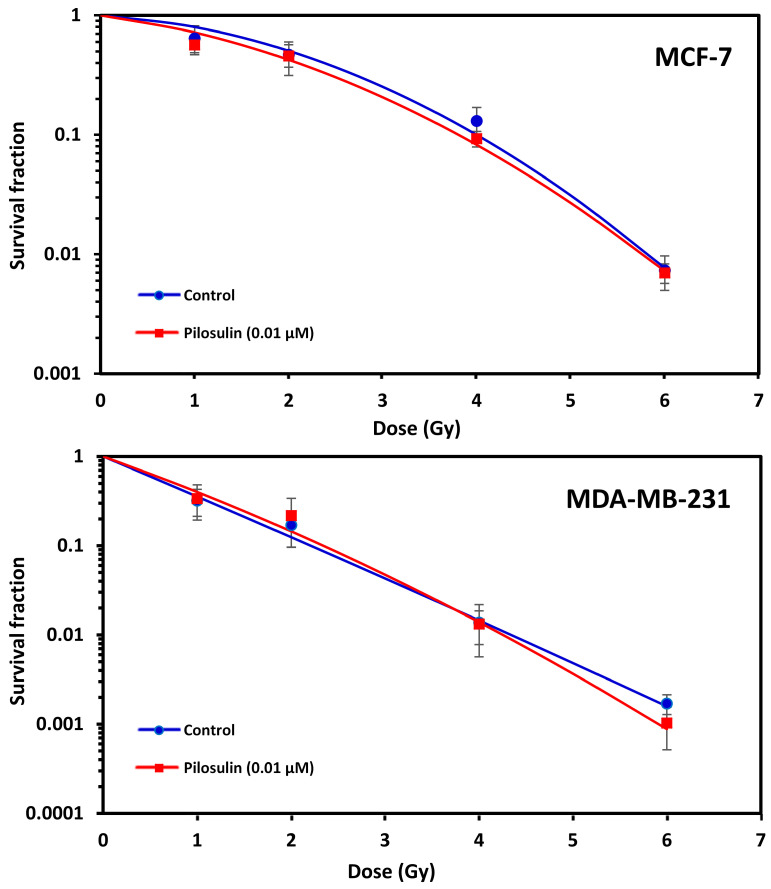
Effect of Pilosulin-3 on radiosensitivity of BC cell lines. Clonogenic survival curves of BC cells following X-ray irradiation with and without Pilosulin-3. Data represent the average of three independent experiments. Error bars represent the SE of the mean.

The ability of Pilosulin-3 to radiosensitize BC cells irradiated after 24 h incubation at the IC25 (0.01 µM) concentration was studied using the clonogenic cell survival curves. As illustrated in Figure 5, both cell lines showed typical cell survival curves where survival decreased exponentially with increasing radiation doses. As expected, MDA-MB-231 was more sensitive to irradiation than MCF-7. The SF2 was 0.12 (95% Confidence Interval (95%CI): 0.10–0.16) in MDA-MB-231 compared to 0.50 (95%CI: 0.42–0.61) in MCF-7 (i.e., ~four-fold difference in radiosensitivity). Incubation with Pilosulin-3 did not clearly enhance the sensitivity of the cell strains to radiation-induced cytotoxicity (SF2 with Pilosulin-3 was 0.14 (95%CI: 0.1–0.20) and 0.43 (95%CI: 0.37–0.49) for MDA-MB-231 and MCF-7, respectively). The DMFs at 10% survival were 0.94 and 1.05 in MDA-MB-231 and MCF-7, respectively.

## 3. Discussion

This study investigated the effect of Pilosulin-3, a commercially available recombinant ant venom, on breast cancer cells in vitro both alone and in combination with radiation treatment. The motivation was to explore its potential as an alternative non-traditional agent to combat cancer, particularly in combination with radiation. While radiation therapy has been effective in treating cancer, it often causes harm to nearby healthy cells [27]. The development of cancer treatments with minimal side effects has been a major focus of research in the last two decades [5]. To address this, researchers have turned to exploring novel components derived from the venom of insects or animals. Ant venom, in particular, has shown promise in its potential cytotoxic effect [13,17].

The RTCA-based assay was used to evaluate the cytotoxic effects of Pilosulin-3 on BC cell lines at various concentrations. Cellular impedance measurements were quantified using the x-CELLigence system, with 10,000 cells seeded per well to achieve optimal cellular density. The CI values for both cell lines increased rapidly during the first 20 h after seeding, corresponding to cell adhesion. For the lowest Pilosulin-3 concentrations (0.001 to 0.005 μM), the CI remained stable or slightly decreased for up to 24 h. However, higher concentrations of Pilosulin-3 (0.01 to 1μM) led to a drop in CI values. Comparing Pilosulin-3 to its analog, Pilosulin-1, which showed cytotoxic activity by inducing hemolysis in human erythrocytes and lymphocytes [28], Pilosulin-1 was found to cause complete lysis at 40 μM and partial lysis at only 1.25 μM, a much higher concentration than used in this study. Additionally, Pilosulin 1 was observed to have similar kinetics but a more potent cytotoxic effect than Mellitin (bee venom) [28]. Lysis of leukocytes takes place within minutes and usually complete, but the results varied by up to five-fold in leukocytes obtained from different individuals [29]. Pilosulin 2 also demonstrated cytotoxic activity, as it can kill more than 50% of proliferating Epstein–Barr virus-transformed B lymphocytes in 5 min at concentrations as low as 15.6 μM (50 μg/mL) [13]. This concentration is also higher than the toxic concentration of Pilosulin-3 (1 μM). However, Pilosulin 2 did not exhibit any hemolytic activity towards red blood cells at concentrations up to 80 μM [28].

The Pilosulin-3’s IC50 (0.5 µM = 1.229 µg/mL) and IC25 (0.01 µM = 0.024 µg/mL) were determined for both BC cell lines. Notably, these values are lower than those reported for other ant venoms with anticancer effects. For instance, the study of fire ant (*Solenopsis invicta*) venom on endothelial cells reported an IC50 of 3 μg/mL [30], while the study of Achmad et al. [31], testing the inhibition of Burkitt’s Lymphoma cell proliferation using ant nest ethyl acetate extract, reported inhibition of cell growth at the lowest concentration of 15.625 µg/mL. The IC50 of Bee (*A. mellifera*) venom on MDA-MB-231 was also higher (6.25 and 3.125 μg/mL) than that observed in the current study [32]. The impedance-based profiles showed that MCF-7 cells were slightly more resistant to Pilosulin-3 compared to MDA-MB-231 cells. Furthermore, the longer the incubation time (24, 48, and 72 h), the greater the inhibition of cell growth.

Pilosulin-3 demonstrated a notable (*p* < 0.05) cytostatic effect, arresting cell cycle progression at G2/M phase in both BC cell lines when used in combination with radiation treatment (4 Gy) at IC50 (0.5 μM) and IC25 (0.01 μM), as shown via flow cytometry (Figure 3). In MDA-MB-231 cells, treatment with 0.5 µM and 0.01 µM of Pilosulin-3 in combination with radiation (4 Gy) arrested the cell cycle at G2/M phase by 30.85% and 31.74%, respectively. MCF-7 cells, on the other hand, were arrested at G2/M phase of the cell cycle at G2/M phase by 45% and 35.7%, respectively. The positive control for stopping the cell cycle at metaphase (G2/M phase) was colcemid, while a high dose of radiation (10 Gy) was used as a second positive control. This interesting finding of cytostatic effect of Pilosulin-3 is in line with studies using venoms of other species, since various venoms derived from scorpion, spider, bee, wasp, and snake were found to inhibit different cancer cells in vitro [23]. Although membranolytic and non-membranolytic mechanisms of action have been suggested, the exact process by which bioactive peptides kill cancerous cells remains unclear [22]. Since many venom-based anticancer peptides non-specifically destroy the plasma membrane, they provide potential therapeutic pathways for tumors that are not responsive to conventional therapy. Furthermore, the demonstration of a cytostatic effect of Pilosulin-3 in combination with radiotherapy may confer synergistic power, irrespective of a radiosensitization potential, to better control tumor growth and improve the combined treatment outcome.

The four main families of Pilosulins, the *M. pilosula* venom, are unique and have low structural homology to other *Hymenoptera* venom peptides [13]. While previous studies had concentrated on the allergenic, antimicrobial, histamine-releasing, and hemolytic activities [17], to the best of our knowledge, this is the first study on the potential use of Pilosulin-3 as an anticancer agent. Over two decades ago, a patent [33] was granted for Pilosulin-1 (and derivatives) as a cell-inhibitor with cytotoxic activity (U.S. patent No. 6,294,649 B1, 2001). In terms of the development of new peptide-based therapeutics as anticancer agents, the evaluation of selectivity is crucial. Recently, many online databases filled with peptide sequences and their biological meta-data have been developed to screen for toxicity using machine learning programs [34]. We have used the novel ENNAACT tool, which employs neural networks for anticancer peptide prediction to classify the activity of Pilosulin-3 [35]. This machine learning tool predicted the peptide to most likely possess anticancer (PROP = 0.997) and non-hemolytic (PROP = 0.176) properties. These in silico results of high selectivity to cancer cells with low toxicity to normal cells are encouraging and lend support to our data to further develop Pilosulin-3 toward becoming a therapeutic anticancer agent. Nevertheless, these results need to be corroborated with experimental confirmation in a variety of healthy cells to validate the machine learning-based calculations.

The flow cytometry apoptosis measurement showed that Pilosulin-3 had no effect on both BC cell lines at the concentration used in this study as a single agent or in combination with radiation (4 Gy) (Figure 4). As a positive control, DMSO largely killed both cells via apoptosis. Compared with another similar study, Samsum ant venom (SAV) treatment at a concentration of 10 ng/mL potently induced apoptosis in MCF-7 cells 24 h after treatment through an IGF-1-dependant pathway and PI3K/AKT and ERK signaling [36]. Using an Annexin V-FITC binding assay and flow cytometric analysis, the latter study found that the percentage of apoptotic cells exhibited a significant quadruple elevation in the presence of SAV (48%) compared to untreated MCF-7 cells (12%). Clonogenic survival curves showed no radiosensitization effect for MCF-7 and MDA-MB-231 cells treated with Pilosulin-3 compared to control (Figure 5). The dose-modifying factor (MDF) at 10% of survival was 1.05 and 0.94, respectively, which clearly does not support the assumption of the potential use of Pilosuiln-3 as a radiosensitizer to enhance cell killing via radiotherapy.

The results of this study lend some support to the growing evidence for the potential use of animal and insect venom-derived peptides as therapeutic agents for cancer treatment, which has gained increasing attention in recent years [24]. However, these peptides still face several challenges that need to be addressed before they can be used in clinical settings. For example, their short half-life and poor oral bioavailability limit their therapeutic efficacy [37]. Therefore, developing strategies to enhance their stability and bioavailability is a crucial step in realizing their potential as effective cancer therapeutics [24]. The current study has contributed to the growing body of knowledge on the anticancer effects of animal venom-derived peptides by investigating the potential of Pilosulin-3 as a therapeutic agent for breast cancer. Although more research is needed to understand the underlying mechanisms of Pilosulin-3’s anticancer effects, the current study provides a foundation for further investigations into its therapeutic potential, comparing it with other animal venoms that may have anticancer effects [13,37].

## 4. Conclusions

Pilosulin-3 exhibited cytotoxicity on MCF-7 and MDA-MB-231 breast cancer cell lines and induced cytostatic effect in combined therapy but it did not enhance the cell killing by radiation. Thus, the use of Pilosulin-3 as a radiosensitizer may be questionable. Although in silico prediction indicated high selectivity as anticancer with low hemolytic activity, the study pinpoints some limitations such as the need for further investigation of the half-life and stability of Pilosulin-3 and the use of clonogenic assays to estimate IC50 and IC25 values. Overall, the study provides preliminary evidence that Pilosulin-3 may have potential as a cytotoxic agent in breast cancer treatment, but further research is needed in order to fully evaluate its efficacy and safety.

## 5. Materials and Methods

### 5.1. Pilosulin-3 Solution

The recombinant peptide Pilosulin-3 ant venom was obtained from (MyBioSource, Inc., San Diego, CA, USA). Pilosulin-3 was dissolved in phosphate-buffered saline (PBS) at a final concentration of 406.8 μM, and it was diluted depending on each experimental protocol. Selectivity of Pilosulin-3 peptide toward normal and cancer cells was conducted in silico using the ENNAACT tool (https://research.timmons.eu/ennaact, accessed on 27 November 2023) [35].

### 5.2. Cell Culture

The breast cancer (MCF-7 and MDA-MB-231) cell lines were obtained from the American Type Culture Collection (ATCC; Manassas, VA, USA). They were cultured in MEM (Minimum Essential Medium) and supplemented with 1% penicillin/streptomycin and 15% fetal bovine serum (FBS). Cells were incubated at 37 °C in a humidified atmosphere containing 5% CO_2_.

### 5.3. Real Time Cell Proliferation Assay

The real-time cell analyzer (RTCA, ACEA Biosciences Inc., San Diego, CA, USA) was used to assess the cell viability and proliferation, as described elsewhere [38]. Briefly, background measurements were taken from the wells by adding 100 μL of the same culture medium to calibrate the plates using the RTCA Software Package 1.2. Cells were seeded in E-Plate 16 (ACEA Biosciences Inc., San Diego, CA, USA) at a density of 10,000/well with fresh medium to a final volume of 180 μL. The plate was left to settle for 30 min at room temperature to ensure that the culture media and E-Plate surface achieve equilibrium before the impedance signals recording. After 24 h, cells were treated with Pilosulin-3 at 200 µL final volume at different concentrations, ranging from 0 (control) to 1 µM. A “blank” control containing only fresh complete medium was also included. Readings were made at 15 min intervals until the end of the experiment (up to 96 h). Electronic readings change as cells that proliferate or die attach or detach from the surface electrodes, thus producing a change in impedance that is calculated via complex mathematical algorithms and plotted as a cell index (CI) value [39]. The CI is an arbitrary unit, the magnitude of which depends on cell number, cell morphology, cell size, and the strength of cell attachment to the substrate coating the plate.

### 5.4. Cell Cycle Analysis

Cells were seeded at a density of 100,000 cells/well in 35 mm cell culture dishes and incubated at 37 °C. The media were removed after 24 h and were replaced with 1 mL fresh media with Pilosulin-3 at 0.01 and 0.5 µM. After 4 h, the cells were irradiated with X-ray doses of 4 Gy using X-RAD 320 Biological Irradiator (Precision X-ray, Madison, CT, USA) at a maximum energy of 320 KVp, 2 mm Al filter, and 1 Gy/min of dose rate. Additional controls were added and consisted of cells treated with colcemid (KaryoMax, Gibco, Thermo Fisher Scientific Inc., Bedford, MA, USA), which prevents spindle formation during mitosis, causing metaphase arrest, cells exposed to 10 Gy high “lethal” radiation dose, and PBS (that was used to dissolve Pilosulin-3). Cells were incubated for 24 h; then, they were trypsnized, washed with ice-cold PBS, centrifuged, and then fixed with 700 µL 70% ethanol. Fixed cells were collected via centrifugation at 500 rpm for 5 min then resuspended in 500 µL PBS containing 10 µg/mL RNase (Thermo Fisher Scientific Inc., Bedford, MA, USA) at 37 °C for 15 min. Cells were stained with 2 mg/mL propidium iodide (PI; Thermo Fisher Scientific Inc., Bedford, MA, USA) at a final concentration of 10 µg/mL and incubated for 15 min at 4 °C in the dark. Flow cytometry analysis has been performed on a FACSCalibur analyzer (BD Biosciences, Franklin Lakes, NJ, USA) and the data were analyzed using CellQuest 3.3 software [40].

### 5.5. Cell Apoptosis Analysis

Cells have been seeded at a density of 100,000 cells/well in a 35 mm cell culture dish and incubated at 37 °C. The media was removed after 24 h and were replaced with 1 mL fresh media with Pilosulin-3 at 0.01 and 0.5 µM. After 4 h, the cells were irradiated with 4 Gy or 10 Gy X-ray doses using X-RAD 320 Biological Irradiator. Additional controls were treated with PBS, 10% DMSO (as a positive control to induce apoptosis), or 10 Gy high radiation dose. After 24 h, the cells were detached and washed twice with cooled PBS. Subsequently, the cells were collected and resuspended in 5 µL Alexa Fluor 488 annexin V “Component A” (Thermo Fisher Scientific Inc., Branchburg, NJ, USA) at 37 °C for 15 min. Then, 400 µL annexin-binding buffer was added, mixed gently, and incubated for 10 min, followed by staining with 2 mg/mL PI. Flow cytometry analysis has been performed on a FACSCalibur analyzer [41].

### 5.6. Clonogenic Survival Assay

Cells were assessed via clonogenic assay as described previously [42]. Briefly, breast cancer cells were plated and treated with IC25 (0.01 µM) of Pilosulin-3 for 24 h before irradiation with either 0, 1, 2, 4 or 6 Gy doses using X-RAD 320 Biological Irradiator. Cells were incubated for 2 weeks to form visible colonies. Cells were fixed and stained with crystal violet for 30 min [43]. Colonies containing at least 50 cells were counted as surviving. Survival data from at least 3 independent experiments were pooled, and survival curves were fitted and analyzed using the linear-quadratic model (LQ). Radiation sensitivity is expressed in terms of the surviving fraction at 2 Gy (SF2) [44]. To evaluate the efficacy of drug treatment, the magnitude of radiosensitization is expressed as a dose modifying factor (DMF), which is defined as the ratio of radiation–doses required to obtain the same level of effect (arbitrarily 10% isoeffect survival) with radiation treatment alone compared to combined treatment with Pilosulin-3 [27,44,45].

### 5.7. Statistical Analysis

Cell index (CI) for real-time dynamic cytotoxicity assessment was calculated automatically by the RTCA Software Package 1.2. Calculation of the normalized cell index (CI %) was carried out by dividing the cell indices at each time point after compound addition by the cell index of the untreated control (0 µM). Comparison between various treatments was carried out using one-way repeated-measures analysis of variance. A *p*-value of <0.5 is considered statistically significant.

## Data Availability

The data presented in this study are available in the manuscript.

## Supplementary Materials

The following supporting information can be downloaded at https://www.mdpi.com/article/10.3390/toxins15120701/s1: Figure S1: Example of cell proliferation results using the Real-Time Cell Analyzer (RTCA) assay. Figure S2: Example of flow cytometry dot-plots results of the effect of Pilosulin-3 on cell cycle in MCF-7 and MDA-MB-231 breast cancer cell lines. Figure S3: Example of flow cytometry histograms results of the effect of Pilosulin-3 on apoptosis induction in MCF-7 and MDA-MB-231 breast cancer cell lines.
