# Supplementary material for: Cytotoxicity and Radiosensitizing Potentials of Pilosulin-3, a Recombinant Ant Venom, in Breast Cancer Cells
Source: Toxins (Basel). 2023 Dec 15;15(12):701. doi: 10.3390/toxins15120701 (PMC10747674; doi:10.3390/toxins15120701)
Supplement: Supplementary file 1 [file toxins-15-00701-s001.zip › toxins-2630025-supplementary.pdf]

Cytotoxicity and Radiosensitizing Potentials of Pilosulin-3, a Recombinant Ant Venom, in Breast Cancer Cells

Reema M. Alzeer, Khaled S. Al-Hadyan, Najla M. Al-Harbi, Sara S. Bin Judia, Rafa S. Almeer and Ghazi A. Alsbeih

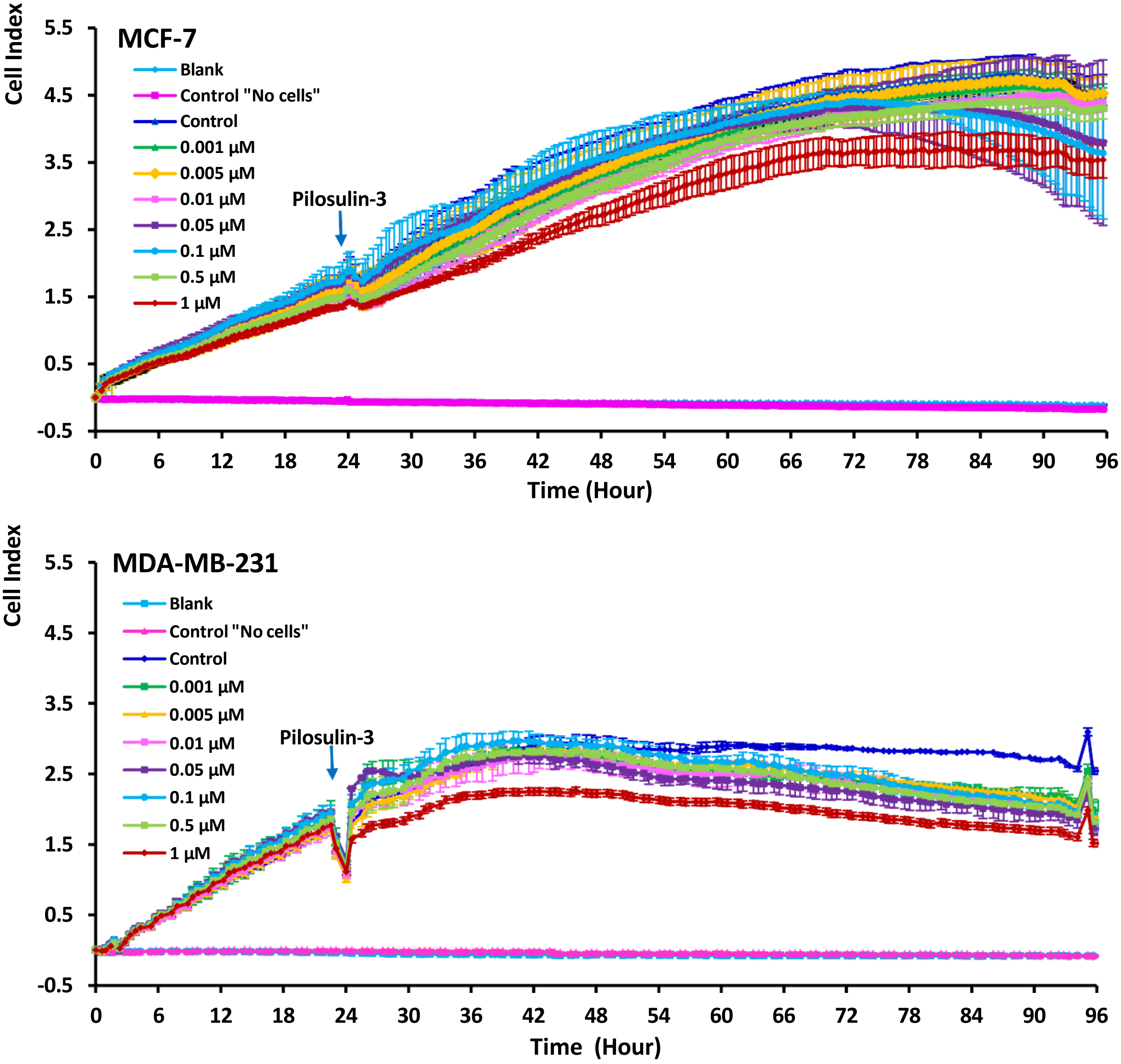

Figure S1. Example of cell proliferation results using the Real-Time Cell Analyzer (RTCA) assay.

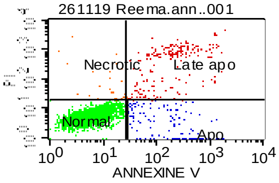

File: 261119 Reema.ann..00
Sample ID: Control
Patient ID: MCF-7

Region	% Gated
Normal	88.65
Apo	4.10
Late apc	6.45
Necrotic	0.83
Total	100.00

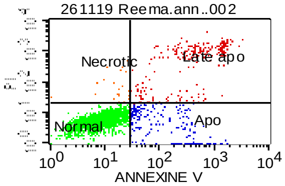

File: 261119 Reema.ann..00
Sample ID: Control +PBS
Patient ID: MCF-7

Region	% Gated
Normal	82.79
Apo	8.85
Late apc	7.49
Necrotic	0.87
Total	100.00

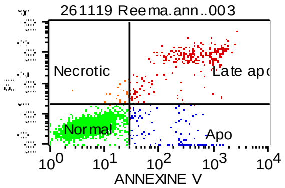

File: 261119 Reema.ann..00
Sample ID: Drug 0.5uM
Patient ID: MCF-7

Region	% Gated
Normal	89.31
Apo	2.95
Late apc	6.98
Necrotic	0.76
Total	100.00

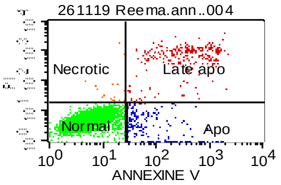

File: 261119 Reema.ann..00
Sample ID: Radiation 4Gy
Patient ID: MCF-7

Region	% Gated
Normal	87.81
Apo	4.66
Late apc	6.72
Necrotic	0.82
Total	100.00

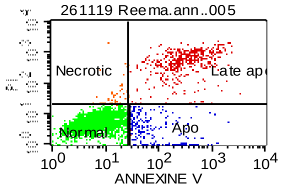

File: 261119 Reema.ann..005
Sample ID: 4 Gy + Drug 0.5uM
Patient ID: MCF-7

Region	% Gated
Normal	80.62
Apo	5.73
Late apc	12.51
Necrotic	1.11
Total	100.00

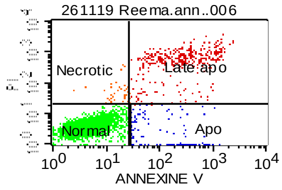

File: 261119 Reema.ann..00
Sample ID: Drug 0.01uM
Patient ID: MCF-7

Region	% Gated
Normal	84.71
Apo	4.06
Late apc	10.13
Necrotic	1.10
Total	100.00

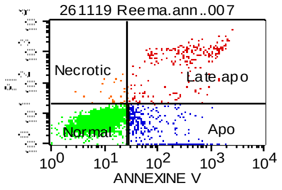

File: 261119 Reema.ann..007
Sample ID: 4 Gy + Drug 0.01uM
Patient ID: MCF-7

Region	% Gated
Normal	85.68
Apo	7.35
Late apc	6.30
Necrotic	0.64
Total	100.00

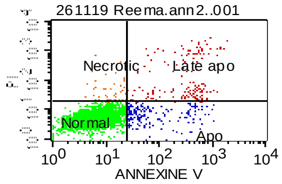

File: 261119 Reema.ann2..00
Sample ID: Control
Patient ID: MDA

Region	% Gated
Normal	82.67
Apo	8.59
Late apc	6.50
Necrotic	2.22
Total	100.00

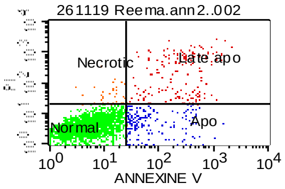

File: 261119 Reema.ann2..00
Sample ID: Control + PBS
Patient ID: MDA

Region	% Gated
Normal	86.50
Apo	6.74
Late apc	5.59
Necrotic	1.14
Total	100.00

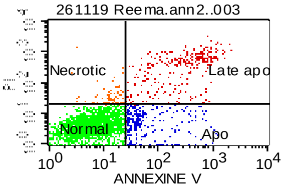

File: 261119 Reema.ann2..00
Sample ID: Drug 0.5uM
Patient ID: MDA

Region	% Gated
Normal	77.39
Apo	9.70
Late apc	10.28
Necrotic	2.61
Total	100.00

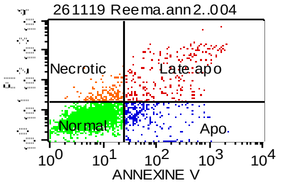

File: 261119 Reema.ann2..00
Sample ID: Radiation 4Gy
Patient ID: MDA

Region	% Gated
Normal	78.65
Apo	8.40
Late apc	7.45
Necrotic	5.62
Total	100.00

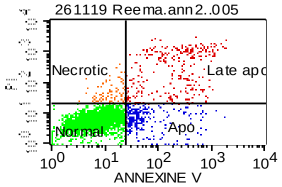

File: 261119 Reema.ann2..00!
Sample ID: 4Gy + Drug 0.5uM
Patient ID: MDA

Region	% Gated
Normal	77.01
Apo	10.03
Late apc	9.41
Necrotic	3.55
Total	100.00

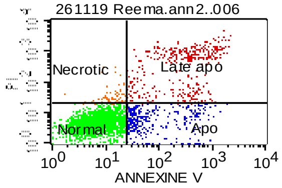

File: 261119 Reema.ann2..00
Sample ID: Drug 0.01uM
Patient ID: MDA

Region	% Gated
Normal	72.61
Apo	11.55
Late apc	13.45
Necrotic	2.45
Total	100.00

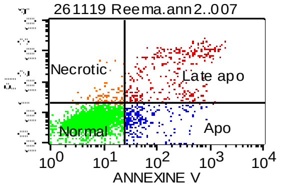

File: 261119 Reema.ann2..007
Sample ID: 4 Gy + Drug 0.01uM
Patient ID: MDA

Region	% Gated
Normal	77.11
Apo	9.26
Late apc	10.68
Necrotic	3.01
Total	100.00

Figure S2. Example of flow cytometry dot-plots results of the effect of Pilosulin-3 on cell cycle in MCF-7 and MDA-MB-231 breast cancer cell lines.

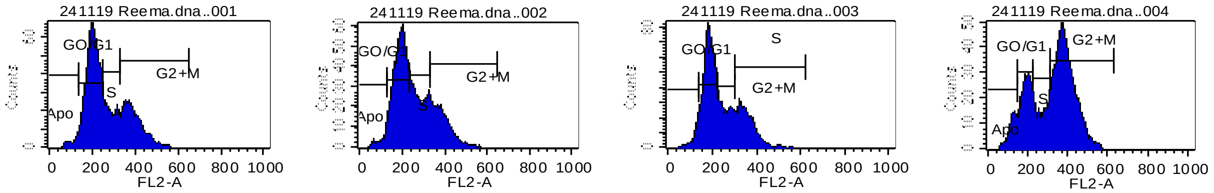

File: 241119 Reema.dna..00
Sample ID: Control
Patient ID: MCF-7

Marker	% Gated
All	100.00
Apo	2.78
GO/G1	55.31
S	16.25
G2+M	25.78

File: 241119 Reema.dna..00
Sample ID: Control +PBS
Patient ID: MCF-7

Marker	% Gated
All	100.00
Apo	3.13
GO/G1	51.43
S	25.75
G2+M	20.23

File: 241119 Reema.dna..00
Sample ID: Drug 0.5uM
Patient ID: MCF-7

Marker	% Gated
All	100.00
Apo	5.04
GO/G1	51.32
S	20.01
G2+M	23.72

File: 241119 Reema.dna..00
Sample ID: 4 Gy Radiation
Patient ID: MCF-7

Marker	% Gated
All	100.00
Apo	6.71
GO/G1	19.60
S	16.25
G2+M	57.61

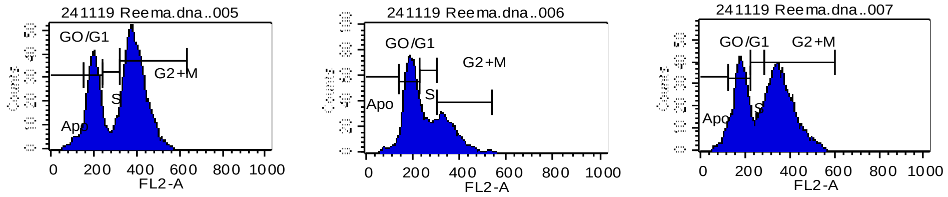

File: 241119 Reema.dna..005
Sample ID: 4 Gy + Drug 0.5uM
Patient ID: MCF-7

Marker	% Gated
All	100.00
Apo	4.45
GO/G1	26.99
S	9.50
G2+M	59.99

File: 241119 Reema.dna..00
Sample ID: Drug 0.01uM
Patient ID: MCF-7

Marker	% Gated
All	100.00
Apo	4.29
GO/G1	50.43
S	18.84
G2+M	27.21

File: 241119 Reema.dna..007
Sample ID: 4 Gy + Drug 0.01uM
Patient ID: MCF-7

Marker	% Gated
All	100.00
Apo	3.67
GO/G1	31.81
S	12.08
G2+M	53.26

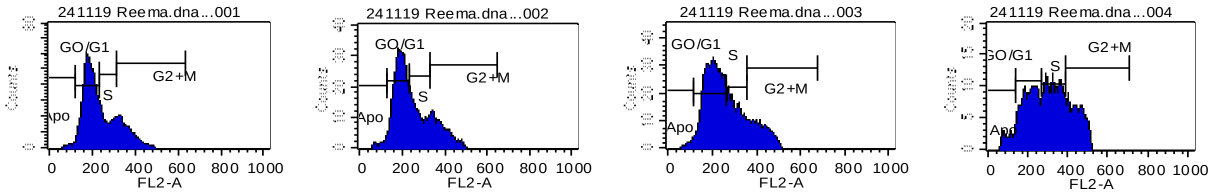

File: 241119 Reema.dna...00
Sample ID: Control
Patient ID: MDA

Marker	% Gated
All	100.00
Apo	1.77
GO/G1	59.78
S	18.87
G2+M	19.73

File: 241119 Reema.dna...00
Sample ID: Control +PBS
Patient ID: MDA

Marker	% Gated
All	100.00
Apo	2.74
GO/G1	56.39
S	23.21
G2+M	18.03

File: 241119 Reema.dna...00
Sample ID: Drug 0.5uM
Patient ID: MDA

Marker	% Gated
All	100.00
Apo	1.41
GO/G1	60.44
S	23.85
G2+M	15.49

File: 241119 Reema.dna...00
Sample ID: 4 Gy Radiation
Patient ID: MDA

Marker	% Gated
All	100.00
Apo	4.80
GO/G1	35.10
S	37.52
G2+M	21.29

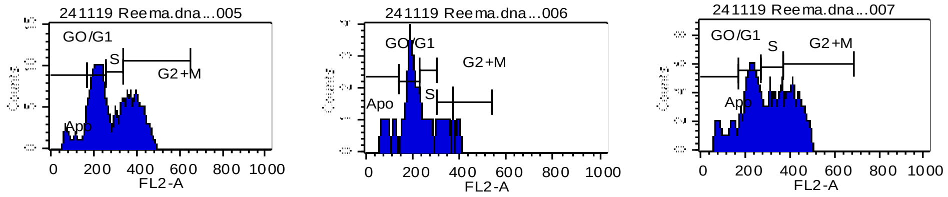

File: 241119 Reema.dna...00
Sample ID: 4 Gy+ Drug 0.5uM
Patient ID: MDA

Marker	% Gated
All	100.00
Apo	9.55
GO/G1	40.61
S	18.08
G2+M	33.04

File: 241119 Reema.dna...00
Sample ID: Drug 0.01uM
Patient ID: MDA

Marker	% Gated
All	100.00
Apo	13.38
GO/G1	51.59
S	16.56
G2+M	18.47

File: 241119 Reema.dna...007
Sample ID: 4 Gy+ Drug 0.01uM
Patient ID: MDA

Marker	% Gated
All	100.00
Apo	11.96
GO/G1	33.12
S	25.89
G2+M	29.73

Figure S3. Example of flow cytometry histograms results of the effect of Pilosulin-3 on apoptosis induction in MCF-7 and MDA-MB-231 breast cancer cell lines.
